# Cyclic voltammetry as a method for determining the viability of seeds: a case study on silver maple (*Acer saccharinum* L.)

**DOI:** 10.1186/s12870-025-07137-x

**Published:** 2025-08-14

**Authors:** Magdalena Trusiak, Danuta Zielińska, Paulina Luśnia, Tomasz Mikołajczyk, Marcin Michalak, Beata P. Plitta-Michalak

**Affiliations:** 1 Department of Forestry and Forest Ecology, Faculty of Agriculture and Forestry, University of Warmia and Mazury in Olsztyn, Plac Łódzki 2, Olsztyn, 10-719 Poland; 2 Department of Chemistry, Faculty of Agriculture and Forestry, University of Warmia and Mazury in Olsztyn, Plac Łódzki 4, Olsztyn, 10-719 Poland

**Keywords:** *Acer saccharinum*, Antioxidant capacity, Cyclic voltammetry, Oxidative stress, Seed aging, Seed viability

## Abstract

**Background:**

Efficient and dependable techniques for determining seed viability are essential in agronomy, forestry, and safeguarding endangered plant species, as seeds represent the most effective way to preserve plant germplasm. Certain seeds can endure preservation for thousands of years, while others may survive only a few weeks. However, all seeds ultimately deteriorate over time during storage. Reactive oxygen species (ROS) and their imbalance with intracellular antioxidants are the primary causes of seed aging and deterioration. Consequently, developing highly effective analytical methods to measure the antioxidant capacity of stored seeds is becoming increasingly critical. This study examines the application of cyclic voltammetry (CV) using a glassy carbon electrode to characterize the antioxidant milieu in aging recalcitrant seeds of *Acer saccharinum* L. This is a versatile electrochemical method that can be easily applied to investigate a broad range of biological matrices because it does not require redox-active reagents to determine the total antioxidant capacity. Instead, it is explicitly based on the electrochemical behavior of antioxidants in samples and their physicochemical properties.

**Results:**

Seed deterioration occurred when *A. saccharinum* seeds with a high moisture content (MC of 45%) were subjected to accelerated aging at 35 °C for up to 14 days. Oxidative stress and antioxidant depletion were monitored by measuring ROS levels, quantifying antioxidants through the Cu^2+^ reduction reaction (CUPRAC-BCA) and CV, and evaluating the glutathione half-cell reduction potential (E_GSSG/2GSH_). Compared with Cu^2+^ reduction measurements, which yielded misleading results, CV appeared to be a more reliable technique for differentiating seeds based on their total antioxidant capacity. CV measurements of 80% methanolic and 1x PBS extracts were highly correlated with seed viability, observed as total germination (*R* = 0.92 and 0.86, respectively, *p* ≤ 0.01 for both solvents).

**Conclusions:**

For the first time, we demonstrated a strong correlation between the CV results on the total antioxidant capacity and viability of seeds. This finding suggests that electrochemical techniques can be a quick and efficient method for evaluating seeds prior to germination, potentially from various species. This method enhances seed viability monitoring, achieving 92% accuracy and showing species-agnostic potential, pending validation in lipid-rich seeds.

**Supplementary Information:**

The online version contains supplementary material available at 10.1186/s12870-025-07137-x.

## Introduction

Seed viability is the most significant factor in seed quality evaluation. Nonviable seeds reduce plantlet production and cause severe economic losses for businesses that rely on germinating seeds, such as forestry and agriculture. On the other hand, the seed banking of rare plant species is an important way to safeguard genetic diversity in the long term. Billions of seeds from wild species are currently stored in hundreds of conservation seed banks worldwide [1]. Therefore, monitoring seed viability is a standard procedure in gene banks and nurseries, carried out to assess the quality of seeds and determine whether regeneration is necessary to prevent the loss of accessions. Balancing an accurate viability assessment against costs presents a challenge that must be resolved to preserve germplasm efficiently [2]. Seeds naturally age and lose their viability during storage, which affects their longevity. The consequence of this process is a delay in germination, followed by a gradual loss of seed germination capacity, ultimately leading to the death of all seeds in the batch. Several factors, including exceeded levels of reactive oxygen species (ROS) imposing oxidative stress, have been linked to seed aging and loss of viability. Indeed, ROS interact with cellular biomolecules, causing severe and potentially lethal damage to proteins, nucleic acids, and lipids [3–7]. The physiological lesions caused by ROS include loss of membrane integrity *via* lipid peroxidation [8], decreased respiration, protein inactivation and degradation due to carbonylation, and genetic damage resulting from DNA strand breaks and oxidation of nucleobases [7, 9]. However, redox reactions are essential for energy conversion in living cells and also regulate responses to environmental changes. ROS play a crucial role as signalling molecules in plant physiology, including dormancy breaking and germination of seeds, and environmental stress responses [10, 11]. Therefore, they are kept in check rather than being fully neutralized or scavenged. Cells possess mechanisms to regulate uncontrolled oxidation and maintain the balance of ROS. The system involves antioxidant enzymes, such as guaiacol peroxidase (POX), catalases (CATs), superoxide dismutases (SODs), and enzymes of the ascorbate-glutathione cycle, including ascorbate peroxidase (APX), dehydroascorbate reductase (DHR), and glutathione reductase (GR), in association with low-molecular-weight antioxidants (LMWAs), e.g., ascorbic acid and glutathione [3, 4, 6, 12, 13]. The last one has been recognized as a predictive indicator of seed longevity [14, 15], but glutathione function extends beyond ROS detoxification, because it acts as an interface between signaling pathways and metabolic reactions that fuel growth and development [13, 16]. Nonetheless, an imbalance between ROS and the antioxidant defense system, whether it is a cause or a consequence of aging, impacts seed viability and can inhibit or postpone germination. As a result, ROS and antioxidants are often used to evaluate the metabolic and physiological status of seeds, thereby aiding in assessing their viability and longevity [3–6, 12, 15, 17–19].

The term “antioxidant” is generally used for classifying molecules that, when present at low concentrations relative to an oxidizable substrate, can suppress, delay, or prevent oxidation of the same [20–23]. Indeed, antioxidants show multifunctional properties, acting as reducing agents (free radical terminators), metal chelators, and singlet oxygen quenchers [21, 22]. Various protocols have been employed to assess the antioxidant capacity of plant samples [20], making it difficult to compare the results due to the different experimental methods used. These methods utilize different techniques, including high-performance liquid chromatography (HPLC) and various spectrophotometric approaches, including enzymatic and nonenzymatic measurements of lipid peroxidation-inhibiting effects, determination of active oxygen species scavenging capability or radical scavenging activity, including DPPH^•^ and ABTS^•+^ radicals, and measurement of the reducing potential of extracts *via* cupric reducing antioxidant capacity (CUPRAC) [20–22, 24–28]. However, the latter approaches require redox reactive reagents since they are based on the sample’s ability to compete for or scavenge a specific ROS or reduce the oxidized form of metal (Cu^2+^ or Fe^3+^) [25, 29, 30], implicating that the particular method may have a different affinity for various antioxidants [22]. On the other hand, electroanalytical approaches for evaluating antioxidant activity are based on the fact that the oxidation potential is directly related to the antioxidant capacity of the dissolved analytes (tested sample), because it depends only on their physicochemical properties. Since these methods do not require the use of redox-active reagents, they represent a valid improvement over other previously reported methods [21, 22, 31, 32]. For example, the inherent analytical capability of cyclic voltammetry (CV) is based on the phenomenon that when an electrode’s potential is more positive (higher), the electrons in the electrode possess lower energy than the electrons in the highest occupied molecular orbital (HOMO) of the analyte. Consequently, electrons spontaneously transfer from the higher-energy HOMO of the analyte to the lower-energy electrode, resulting in oxidation [31, 33, 34]. Therefore, the lower the oxidation potential measured for the analyte (i.e., sample extract), the stronger the electron-donating (antioxidant) ability of the tested sample. Consequently, the CV tracing (voltammogram) indicates the ability of the analyte to donate electron(s) around the potential of the anodic wave, reflecting the total antioxidant capacity of the sample composed of one or more components. Thus, CV is an alternative to traditional spectrophotometric techniques, serving as a convenient method for the rapid quantitation of total antioxidant capacity without requiring specific determination of each component or the use of additional redox-active reagents [29, 31, 34].

To date, CV has been used to characterize phylogenetic tree based on the voltammetric fingerprints of polyphenolic components in leaves of *Acer* species [35]. However, the most successful and standard application of the technique has been in determining the antioxidant capacity of blood plasma, vegetable oils, milk, orange juice, wines, and winemaking byproducts, as well as selected active compounds or seed extracts of i.e. black cumin (*Nigella sativa* L.) [36], grape (*Vitis vinifera* L.) [37], bitter lupin (*Lupinus angustifolius* L.) [38] or sea bean (*Dioclea reflexa* Hook F.) [39], primarily for assessment of their pro-health or antifungal properties. However, these studies focused on processed plant extracts, neglecting intact plant matrices, where lipid peroxidation may confound redox-active reagents [40, 41]. Therefore, while CV has proven effective in food science, its application to seed viability remains unexamined, particularly for recalcitrant seeds that require real-time assessment of antioxidant capacity. To the best of our knowledge, the voltammetric technique has never been applied to study the antioxidant capacity in aging seeds. The current work aims to demonstrate the potential of CV as a new, fast screening tool for predicting seed viability and longevity.

For our research, we chose non-dormant seeds of *Acer saccharinum* L. (silver maple), which belong to the recalcitrant post-harvest category and are known for the significant storage challenges they present. These seeds are susceptible to water loss below 30% of moisture content (MC) and cannot withstand standard storage conditions at −18 °C due to mechanical damage caused by ice nucleation, metabolism-related damage, and denaturation of macromolecules [4, 12, 17]. As a result, their short lifespan limits storage to less than 18 months at −3 °C [42]. This characteristic makes *A. saccharinum* a valuable model for studying the factors that cause viability loss in recalcitrant species. In the current study, moist seeds were exposed to accelerated aging conditions at 35 °C.

## Materials and methods

### Preparation of research material and germination of seeds

Samaras of *A. saccharinum* L. were collected from trees in Olsztyn, north-eastern Poland (53.7553° N, 20.4561° E). Dr. M. Michalak, Dr. B. Plitta-Michalak, and MSc. M. Trusiak undertook the formal identification of the species and collected samples. This research examines a non-endangered plant species, and local legislation permits the collection of samaras for scientific purposes without requiring legal permission. The voucher specimen has not been deposited in any publicly available herbarium. The MC of samaras and seeds (Supplementary Data 1) was assessed by drying each entity at 103 ± 2 °C for 18 h (four replicates of five entities), and the results are presented in Table 1. For accelerated aging conditions, samaras were placed in sealed bags in an incubator at 35 °C for 1, 4, 10, or 14 days. The germination test was conducted using five biological replicates, each consisting of 50 seeds as previously reported [12].

**Table 1:** Moisture content (MC) of samaras, seeds, and wings of *Acer saccharinum* L.

Storage time (days)	Samaras	Seeds	Wings
0	47.22% ± 1.45%	54.67% ± 2.05%	19.01% ± 1.64%
1	46.25% ± 1.98%	53.42% ± 2.35%	22.79% ± 0.61%
4	44% ± 1.1%	48.87% ± 1.08%	30.41% ± 1.06%
10	44.56% ± 1.33%	48.08% ± 1.04%	35.46% ± 2.19%
14	44.76% ± 1.24	50.51% ± 0.94%	37.43% ± 2.40%

### Measurement of antioxidant capacity using spectrophotometry

The antioxidant capacity (Cu^2+^-AC) was measured using a commercially available Total Antioxidant Capacity Assay kit (Abcam, Cambridge, UK) based on Cu^2+^ reduction (CUPRAC-BCA). Cu^+^ interacts with bicinchoninic acid (BCA), forming a violet chelate complex. Five seeds or embryonic axes in four biological replicates were used in each experiment. The material was homogenized in liquid nitrogen (LN), and samples were extracted in 5 mL (seeds) or 0.5 mL (embryonic axes) of ice-cold 1x phosphate-buffered saline (PBS) pH 7.4, followed by centrifugation at 4 °C for 10 min at 1600 *×g*. The absorbance of the samples (10 µL) was measured at λ 570 nm according to the manufacturer’s instructions using an Infinite M200 Pro plate reader (Tecan, Männedorf, Switzerland). Measurement at this wavelength prevents interference from plant pigments [25]. The antioxidant capacity was calculated based on the Trolox equivalent antioxidant capacity. Calculations were performed based on standard curves for a 0–0.02 mM Trolox solution (*R*^2^ = 0.99). The results are expressed as Trolox equivalent capacity per gram of tissue dry weight (mM g^−1^ DW). For non-protein antioxidant capacity (Cu^2+^-NPAC), samples were diluted 1:1 with Protein Mask provided by the manufacturer. The Protein Mask prevents Cu^2+^ reduction by protein, enabling the analysis of only the LMWAs.

### ROS detection

ROS levels were quantified in seeds using the fluorogenic dye 2′,7′-dichlorodihydrofluorescein diacetate (H_2_DCFDA; Invitrogen, Waltham, MA, USA) as previously reported [43]. Four replicates, each comprising five seeds, were analyzed. Seeds were homogenized in LN, and samples were extracted in 5 mL of ice-cold 1x PBS. The mixture was incubated on ice in the dark with agitation at 650 rpm for 10 min, followed by centrifugation at 4 °C for 10 min at 10,000 ×g. A total of 10 µL of extract was mixed with 90 µL of H_2_DCFDA at a final concentration of 10 µM and incubated in the dark for 10 min. The fluorescence was measured at an excitation wavelength of 492 nm and an emission wavelength of 525 nm using an Infinite M200 PRO plate reader (Tecan, Männedorf, Switzerland). The results are expressed as relative fluorescence units per gram of tissue dry weight (RFU g^−1^ DW).

### Detection of oxidized and reduced glutathione

Oxidized and reduced glutathione were measured using the Quantification kit for oxidized and reduced glutathione based on the reaction of GSH and GSSG with DTNB (Sigma‒Aldrich, Darmstadt, Germany) according to the manufacturer’s protocol. Five seeds were homogenized in LN and extracted in 2 mL of 5% 5-sulfosalicylic acid (SSA). The mixture was subsequently vortexed for 15 s, incubated in the dark at room temperature for 5 min with agitation at 650 rpm, and centrifuged at 8000 × g for 10 min at 4 °C. The supernatant was transferred to a new tube, and the SSA concentration was reduced to 0.5%. The samples (40 µL) and the GSH and GSSG standards were added to a microplate. To determine the GSSG content, the masking agent that binds to GSH was added to some of the samples. After one hour of incubation at 37 °C, 20 µL of coenzyme or enzyme solution was added to each well. The GSH content was determined by subtracting the GSSG concentration from the total glutathione (G_total_) concentration. The GSH and GSSG concentrations were calculated based on standard curves for (0.5–50 µM, *R*^*2*^ = 0.99) and GSSG (0.5–25 µM, *R*^*2*^ = 0.99) solutions. The results are expressed as GSH and GSSG per gram of tissue dry weight (µM g^−1^ DW). GSSG and G_total_ were detected spectrophotometrically at a wavelength of 415 nm. The GSH content was quantified using the following equation: GSH = G_total_ − GSSG × 2. The glutathione half-cell reduction potential (E_GSH/2GSSG_) was calculated according to the Nernst equation [44].

### Preparation of seed extract for cyclic voltammetry

Five seeds in at least four biological replicates were homogenized in LN and extracted with 5 mL of 80% methanol/water (v/v) or ice-cold 1x PBS. For the methanolic extract, the mixture was vortexed for 30 s and then sonicated for 30 s. This step was repeated three times. The mixture was subsequently incubated in the dark for 18 h at room temperature with agitation at 650 rpm and centrifuged for 5 min at 1600 *×g*. For the 1x PBS extract, the mixture was prepared identically to the CUPRAC-BCA (Cu^2+^-AC and Cu^2+^-NPAC) mixtures, which were measured spectrophotometrically. The mixture was incubated on ice in the dark with agitation at 650 rpm for 10 min, followed by centrifugation at 4 °C for 10 min at 1600 *×g*. For each tested condition, three to four independent extractions were carried out. The extracts were used for CV analysis immediately.

### Assessing antioxidant capacity using cyclic voltammetry

A SP-240 potentiostat (BioLogic, France) was used for the voltammetric experiments. A conventional three-electrode system (a) a glassy carbon working electrode (BAS MF-2012), (b) an Ag/AgCl (3 M KCl) electrode as a reference electrode, and (c) a platinum wire as a counter electrode was used in the study as reported earlier [45, 46] (Supplementary Data 2). Before initial use and after measurements of each experimental variant, the working electrode underwent electrochemical cleaning in 0.5 M sulfuric acid until a stable voltammogram profile was achieved, typically after about 10 cycles, against the reference electrode [47]. Before each use, the surface of the glassy carbon electrode was carefully polished with 0.05 μm alumina paste, ultrasonically rinsed in deionized water, and washed with methanol. This procedure was repeated after each cycle. Cyclic voltammetry experiments were performed with (a) 80% methanol/water extracts (CV-MeOH) mixed with 0.2 M sodium acetate‒acetic buffer (pH 4.5) or (b) 1x PBS extracts (CV-PBS) mixed with fresh 1x PBS to a final volume of 10 ml. 80% methanol was selected for its efficacy in extracting LMW polyphenols [48], while PBS mimics physiological conditions for redox-active proteins [49]. The samples were transferred into a glass electrochemical cell. The voltammograms were recorded immediately after the glassy carbon electrode was inserted into the solution, minimizing the adsorption of antioxidants on the electrode surface prior to the run. All measurements were conducted at room temperature. The cyclic voltammogram scans were recorded from − 0.1 to 1 V at a scan rate of 0.1 V·s^−1^. Owing to the capacitive behavior of the electrode, CV requires the recording of the background voltammograms for sample-free buffer-solvent to obtain the background current (blank solution), which was subtracted from the sample. Calculations were made based on standard curves for 0–2.5 mM Trolox solutions for the 80% methanolic mixture (*R*^*2*^ = 0.99) and 0–1 mM Trolox for the 1x PBS mixture (*R*^*2*^ = 0.99). The results are expressed as mM Trolox equivalent per g^−1^ dry weight (DW).

### Statistical analysis

R software (R Core Team 2020) was used for the statistical analyses and graphical visualization of the data. The normality of the data was assessed using the Shapiro–Wilk test. For data that did not follow a normal distribution, the most suitable transformation methods were applied. The Box-Cox transformation was used for total glutathione levels, the Yeo-Johnson transformation for GSH levels, the logit transformation for antioxidant capacity in embryonic axes measured by CUPRAC-BCA, and the arcsine transformation was applied to ROS levels and to antioxidant capacity in seeds measured by CUPRAC-BCA.

The effect of storage time on total antioxidant capacity (CV-MeOH and CV-PBS), antioxidant capacity (Cu^2+^-AC; separate in seeds and embryonic axes), non-protein antioxidant capacity (Cu^2+^-NPAC; separate in seeds and embryonic axes), levels of ROS, GSH and GSSG, the total amount of glutathione (G_total_), and germination mean time (MT) were evaluated using a linear model. A separate one-way ANOVA was conducted for every analysis. The data were subsequently tested for significance (*P* < 0.05) by Duncan’s *post-hoc* multiple range test. A generalized linear model (GLM) with a binomial distribution was used to evaluate the effect of storage on germination (G). Then, the data were tested for significance (*P* < 0.05) using Duncan’s *post-hoc* multiple range test. Spearman’s rank was used for correlation analysis. Principal component analysis was applied to the correlations of germination (G), the total antioxidant capacity (CV-PBS and CV-MeOH), the levels of ROS, GSH and GSSG, the total amount of glutathione (G_total_), antioxidant capacity in seeds (Cu^2+^-AC), non-protein antioxidant capacity in seeds (Cu^2+^-NPAC), and the level of E_GSSG/2GSH_. Principal component analysis was conducted on scaled data.

## Results

### Viability of silver maple seeds: total germination capacity and mean germination rate

**Fig. 1:**
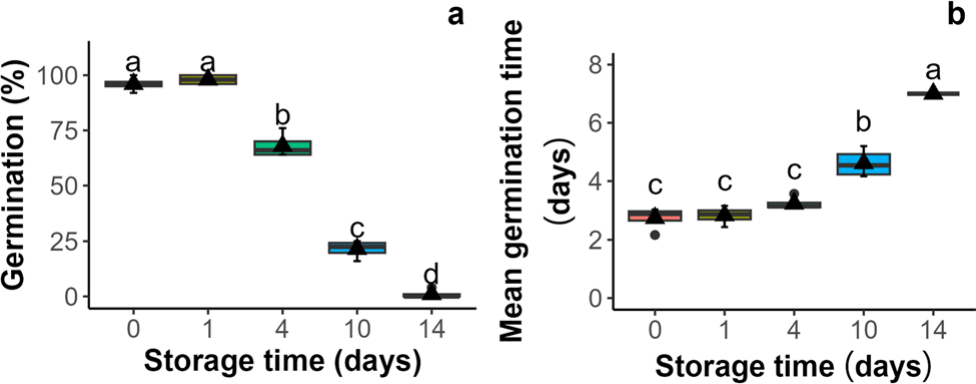
The effect of storage under accelerated aging conditions (45% MC, 35 °C) on total germination (**a**) and germination time (**b**) of *Acer saccharinum* L. seeds. Values labeled with different letters are significantly different at *P* ≤ 0.05, based on Duncan’s multiple range test. Lines denote the median, black triangles represent the mean values, boxes represent the 25^th^ to 75^th^ percentiles, and whiskers represent the 5^th^ to 95^th^ percentiles

The viability of *A. saccharinum* seeds was assessed by evaluating the total germination capacity and mean germination rate [50] of both control seeds and those subjected to accelerated aging for up to 14 days. For the control seeds and those stored for one day, the germination time did not differ significantly (Fig. 1a). After four days of aging, germination significantly decreased from 96% in the control to 68%. Further storage for 10 and 14 days significantly reduced germinability to 21.5% and 1%, respectively. A significant delay in germination time was observed after 10 days, and after 14 days of aging, it increased by 2.5 times (Fig. 1b).

### Assessment of the redox milieu in seeds and embryonic axes

**Fig. 2:**
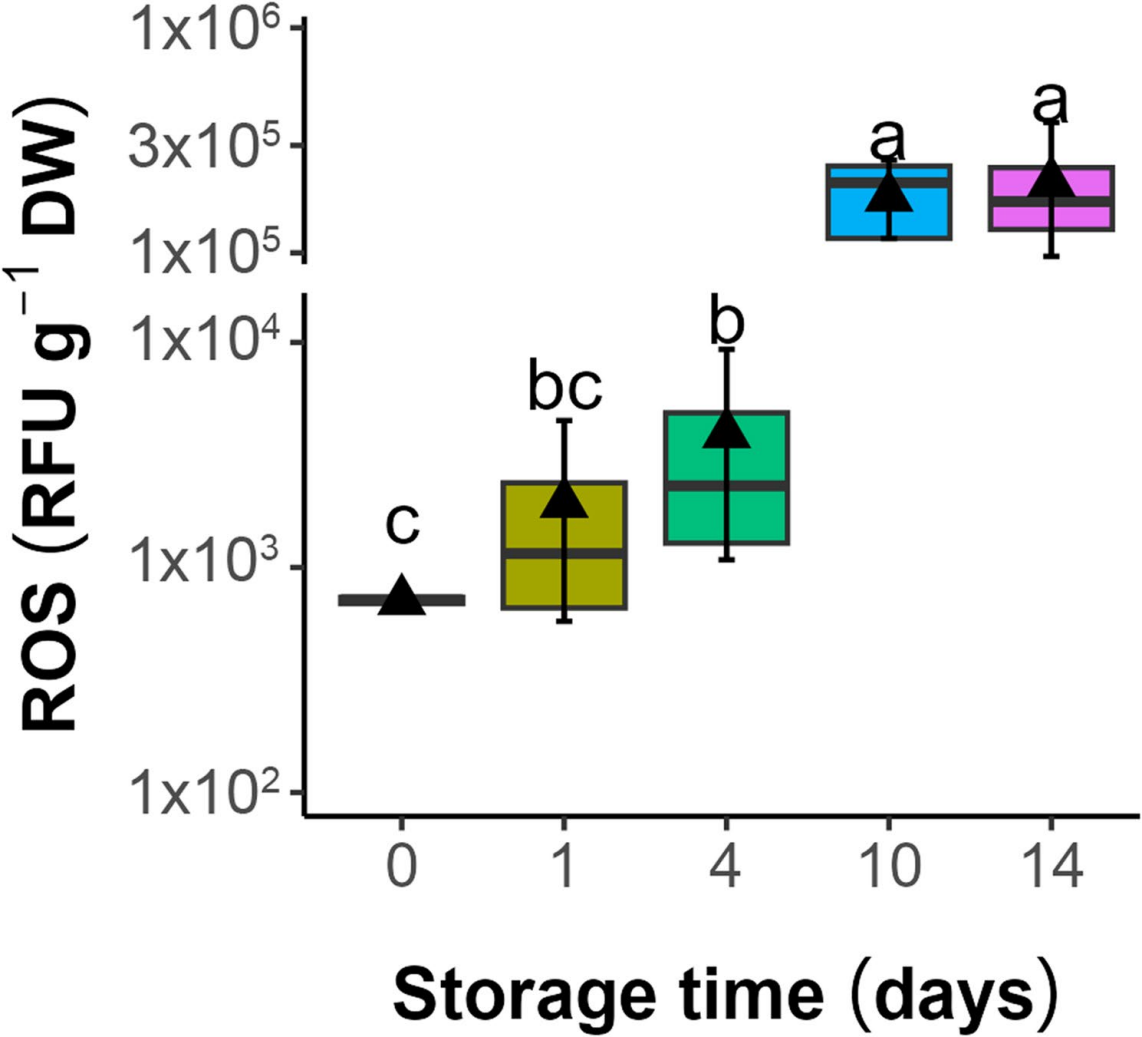
Influence of storage under accelerated aging conditions (45% MC, 35 °C) on the ROS levels in seeds of *Acer saccharinum* L. Lines denote the median, black triangles represent the mean values, boxes represent the 25^th^ to 75^th^ percentiles, and whiskers indicate the 5^th^ to 95^th^ percentiles

The effect of accelerated aging on the ROS levels in seeds was evaluated using the H_2_DCFDA assay (Fig. 2). The treatment resulted in increased ROS levels (1920 and 3916 RFU g⁻¹ DW after 1 and 4 days of aging, respectively), with a notable ROS surge in seeds subjected to accelerated aging for 10 and 14 days (ca. 176 × 10³ and 204 × 10³ RFU g⁻¹ DW, respectively).

**Fig. 3:**
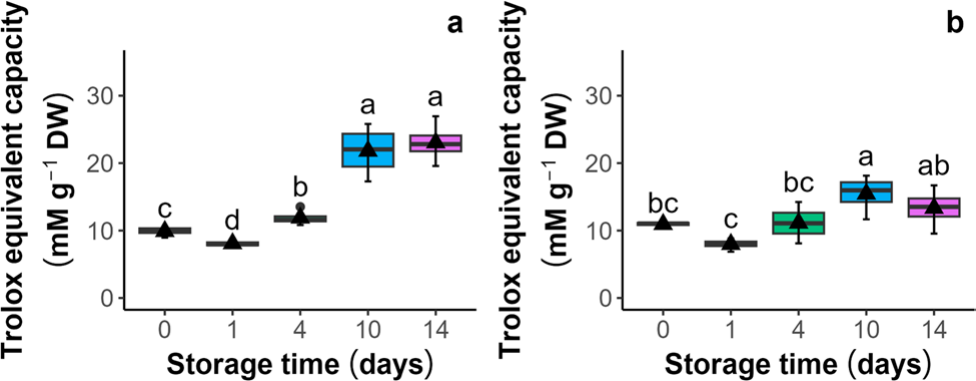
Influence of storage under accelerated aging conditions (45% MC, 35 °C) on the antioxidant capacity (**a**) and the non-protein antioxidant capacity (**b**) of seeds of *Acer saccharinum* L. measured by the Cu^2+^ reduction potential assay (CUPRAC-BCA). Lines denote the median, black triangles represent the mean values, boxes represent the 25^th^ to 75^th^ percentiles, and whiskers represent the 5^t^^h^ to 95^th^ percentiles

**Fig. 4:**
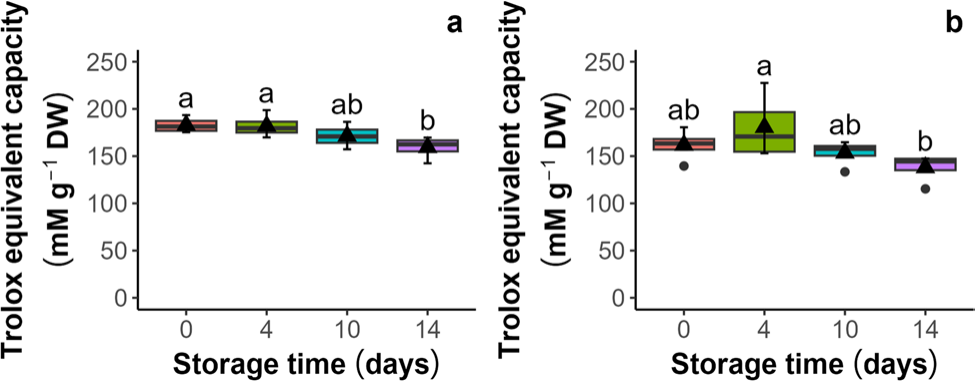
Influence of storage under accelerated aging conditions (45% MC, 35 °C) on the antioxidant capacity (**a**) and the nonprotein antioxidant capacity (**b**) of embryonic axes isolated from seeds of *Acer saccharinum* L. measured by the Cu^2+^ reduction potential assay (CUPRAC-BCA). Lines denote the median, black triangles represent the mean values, boxes represent the 25^th^ to 75^th^ percentiles, and whiskers represent the 5^th^ to 95^th^ percentiles

Antioxidant capacity was evaluated by both the antioxidant capacity (Cu^2+^-AC) and the non-protein antioxidant capacity (Cu^2+^-NPAC) by the Cu^2+^ reduction spectrophotometric assay in the seeds and embryonic axes. In seeds (Fig. 3a and b), both parameters increased, with the highest Cu^2+^-AC levels (21 and 23 mM g⁻¹ DW) and Cu^2+^-NPAC levels (15.4 and 13.3 mM g⁻¹ DW) observed after 10 and 14 days of aging, respectively. Paradoxically, the increase in antioxidants in dead seeds probably indicates Cu^+^-catalyzed decomposition of lipid peroxides [41], highlighting that the CUPRAC-BSA assay is not suitable for lipid-rich systems. In embryonic axes (Fig. 4a and b) isolated from seeds, the trend of changes was reversed, indicating that the levels of both Cu^2+^-AC and Cu^2+^-NPAC decreased from *c.a* 182 and 161 mM g⁻¹ DW, respectively, reaching their lowest points after 14 days (*c.a* 150 and 130 mM g⁻¹ DW, respectively).

**Fig. 5:**
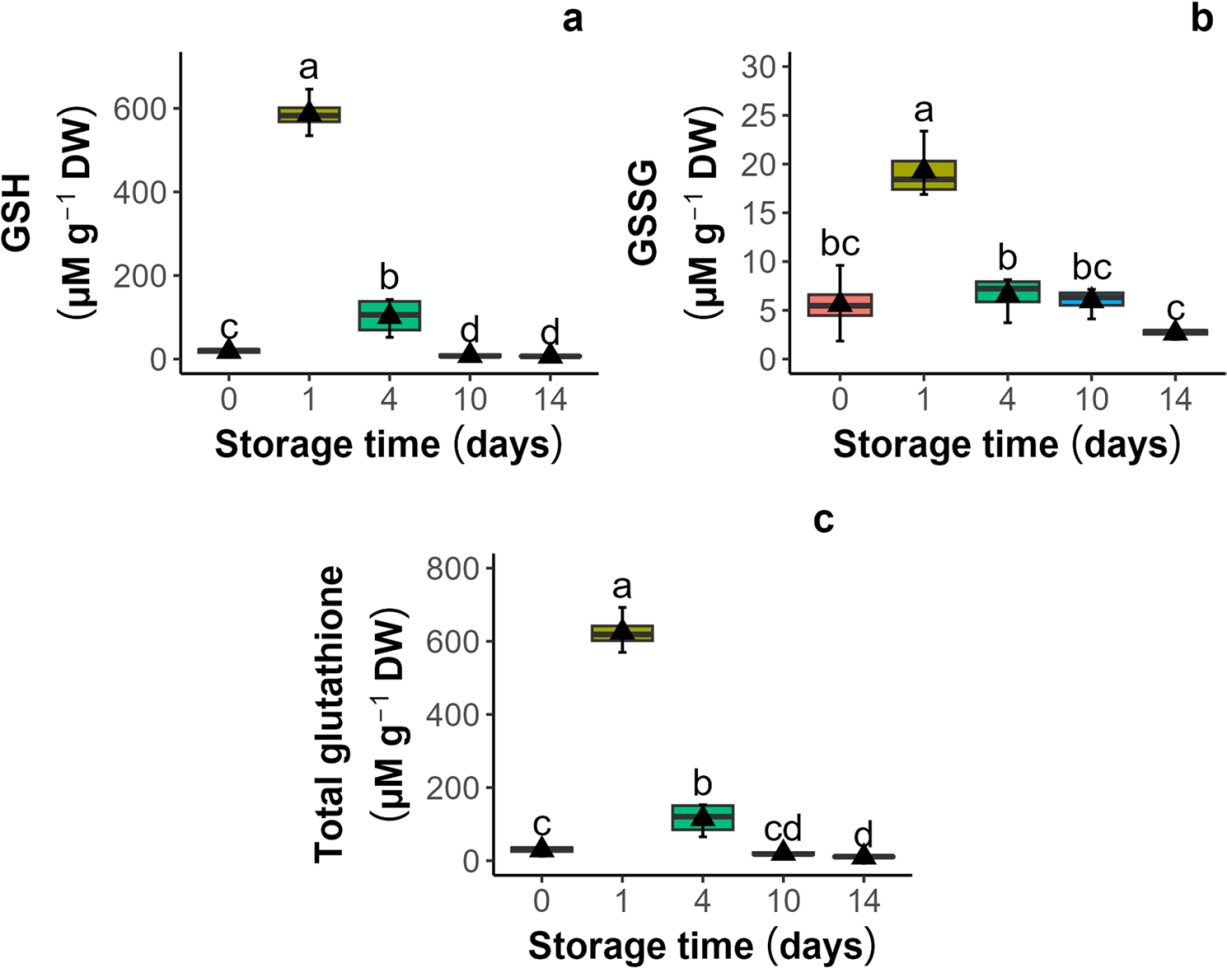
Changes in glutathione levels in *Acer saccharinum* L. seeds subjected to accelerated aging (45% MC, 35 °C): (**a**) reduced glutathione (GSH), (**b**) oxidized glutathione (glutathione disulfide; GSSG), (**c**) total glutathione pool (G_total_). Lines denote the median, black triangles represent the mean values, boxes represent the 25^th^ to 75^th^ percentiles, and whiskers represent the 5^th^ to 95^th^ percentiles

**Fig. 6:**
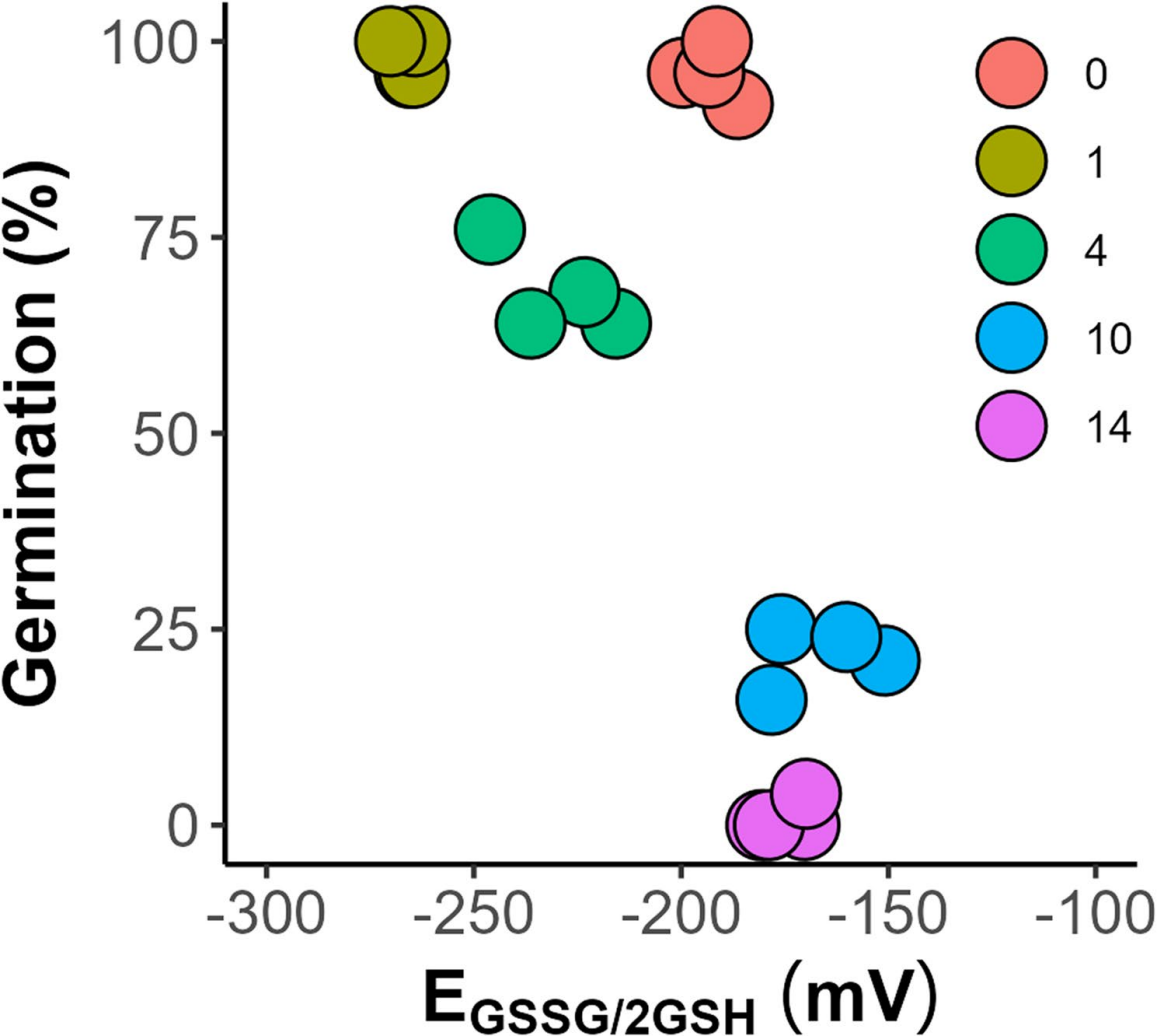
The half-cell reduction potential (E_GSSG/2GSH_) calculated for *Acer saccharinum* L. seeds subjected to accelerated aging (45% MC, 35 °C) using the Nernst equation

The aging process of the seeds was tracked by monitoring the concentrations of GSH and GSSG (Fig. 5a and b). In the control group of unaged seeds, the GSH concentration was 18.2 µM g⁻¹ DW, while the GSSG concentration was 5.5 µM g⁻¹ DW. After one day of aging, both compounds spiked with 588 and 19.2 µM g⁻¹ DW, respectively. However, after further storage at 35 °C for 14 days, the levels of GSH and GSSG in dead seeds decreased to 6.99 and 2.65 µM g⁻¹ DW, respectively. The changes in the total glutathione pool (G_total_) (Fig. 5C) corresponded to changes in GSH and GSSG, which significantly decreased to 9.97 µM g⁻¹ DW after 14 days of accelerated aging, which was 3 times less than that in the control seeds (29.4 µM g⁻¹ DW) and 62 times greater than that after one day of storage when the GSH outburst was observed. The half-cell reduction potentials of the glutathione/glutathione disulfide redox couple (E_GSSG/2GSH_) were calculated according to the Nernst equation [44] at an assumed cellular pH of 7.4 (Fig. 6). In this study, seed viability decreased with increasing positive values of E_GSSG/2GSH_ up to average values of −164 and − 173 mV for seeds stored for 10 and 14 days, respectively.

### Analysis of antioxidant capacity using CV

**Fig. 7:**
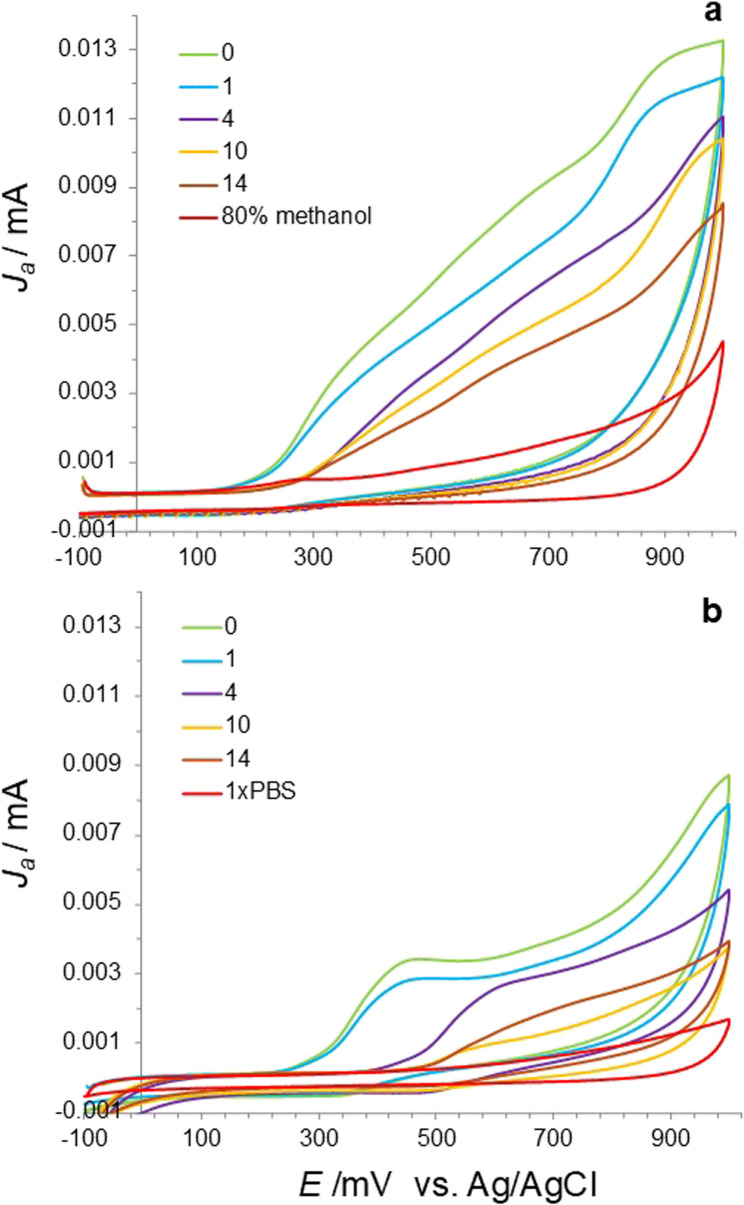
Selected cyclic voltammograms of 80% (v/v) methanol/water extracts (**a**) and 1x PBS extracts (**b**) from *Acer saccharinum* L. seeds subjected to accelerated aging (45% MC, 35 °C) recorded from − 0.1 to 1 V at a scanning rate of 0.1 V·s^−1^

**Fig. 8:**
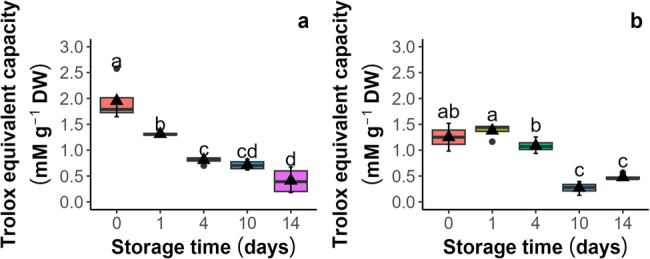
Total antioxidant capacity in seeds of *Acer saccharinum* L. subjected to accelerated aging (45% MC, 35 °C) assessed using the cyclic voltammetry method expressed as mM Trolox equivalents g^−1^ DW; (**a**) CV-MeOH (80% (v/v) methanol/water extracts), (**b**) CV-PBS (1x PBS extracts)

This study evaluated the antioxidant capacity of two extract types, 80% methanolic (CV-MeOH) and 1x PBS (CV-PBS), derived from *A. saccharinum* seeds, utilizing the CV method. The cyclic voltammograms showed a broad anodic wave without distinct peaks, indicating the presence of various antioxidants, each donating electrons, which reflects the overall oxidation potential of the tested sample. The lowest values of potential (E) are, the greater facility or strength of molecules in the mixture for the electrodonation and, thus, to act as stronger antioxidants [27, 31, 51]. No cathodic wave was observed when the scan direction was inverted, indicating the irreversibility of the oxidation of the extracts (Fig. 7a and b). The electrochemical parameter used for quantifying the antioxidant capacity was the total anodic charge (anodic current area; AUC) [29], which was calculated by integrating the cyclic voltammograms between potentials of 0.1 and 1.0 V. The voltammograms demonstrated a gradual decline in the antioxidant capacity of the 80% methanolic extract (CV-MeOH; Fig. 8a), which revealed a nearly fivefold decrease from 1.95 to 0.4 mM Trolox equivalents g^−1^ DW as the duration of seed aging increased. In the 1x PBS buffer extract (CV-PBS; Fig. 8b), the lowest values for antioxidant capacity, which were not significantly different from each other, were recorded after 10 and 14 days of aging, with values of 0.27 and 0.47 mM Trolox equivalents g^−1^ DW, respectively. Furthermore, no notable differences emerged between the control seeds and those aged for one day, as the mM Trolox equivalents g^−1^ DW were 1.25 and 1.37, respectively.

### Correlation and principal component analysis

**Fig. 9:**
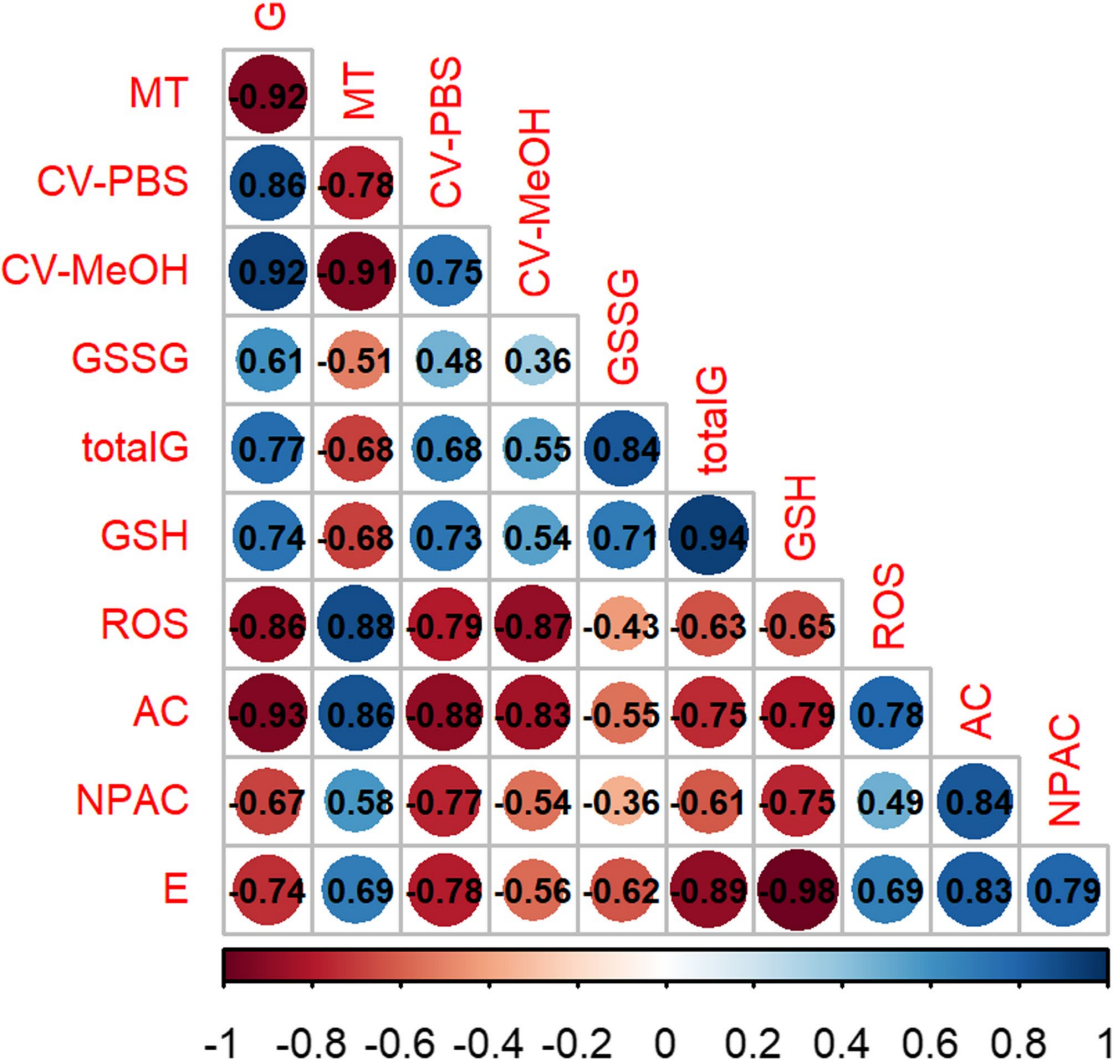
Spearman correlation coefficients between the means of oxidative stress measurements (ROS), total germination (G) and germination time (MT), seed antioxidant capacity (Cu^2+^-AC), seed non-protein antioxidant capacity (Cu^2+^-NPAC), levels of glutathione disulfide (GSSG) and glutathione (GSH), total amount of glutathione (GSH + GSSH; G_total_), half-cell reduction potential (E_GSSG/2GSH_; E), total antioxidant capacity of methanolic extract (CV-MeOH), and total antioxidant capacity of 1x PBS extracts (CV-PBS) measured for *Acer saccharinum* L. seeds subjected to accelerated aging (45% MC, 35 °C). The size of the circles represents the level of correlation (r), and larger circles indicate that a given trait correlates at a higher level. The blue color indicates positive correlations, and the red color indicates negative correlations. *p* ≥ 0.01

A very strong positive correlation was observed between the total germination of seeds (G) and the total antioxidant capacity of the methanolic (CV-MeOH) and 1x PBS (CV-PBS) extracts (Fig. 9). A lower correlation between germinability and PBS extracts may result from protein-antioxidant interactions that obscure redox signals, while an 80% methanolic solution extracts free phenolics more efficiently [52]. A strong negative correlation was observed between seed germination and the ROS level. A strong negative correlation was also detected between total germination and Cu^2+^-AC. However, this result is considered misleading and should be discussed further.

**Fig. 10:**
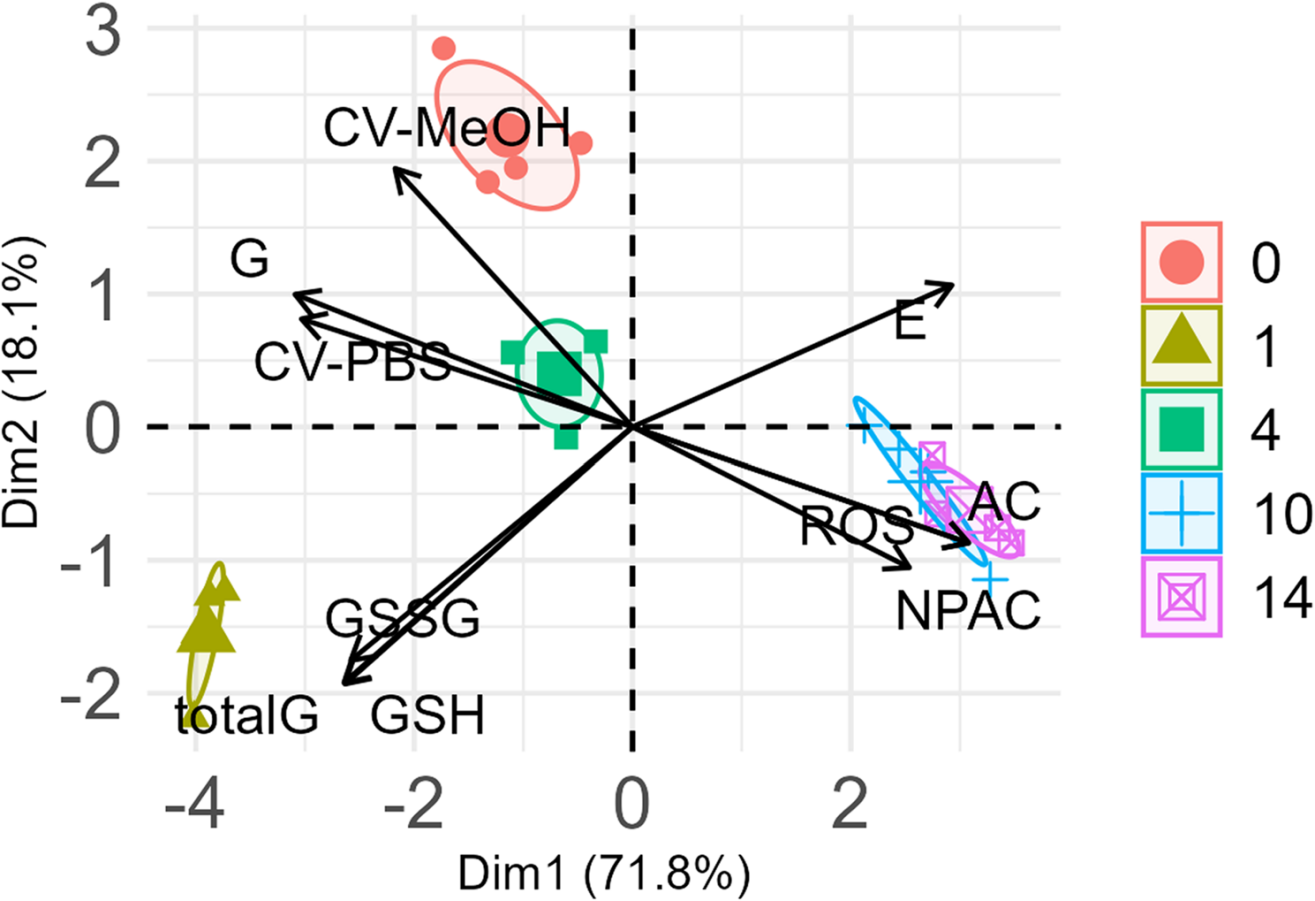
Results of principal component analysis (PCA) of the correlations of total germination (G), germination mean time (MT), ROS level (ROS), antioxidant capacity (Cu^2+^-AC), non-protein antioxidant capacity (Cu^2+^-NPAC), total antioxidant capacity of methanolic extracts (CV-MeOH), total antioxidant capacity of 1x PBS extracts (CV-PBS), levels of glutathione disulfide (GSSG) and glutathione (GSH), total amount of glutathione (GSH + GSSH; G_total_), and half-cell reduction potential (E_GSSG/2GSH_; E) for *Acer saccharinum* L. seeds subjected to accelerated aging (45% MC, 35 °C). Ellipse confidence level = 0.95

Based on principal component analysis, *A. saccharinum* seeds were grouped into four clusters, clearly separating seeds with low viability (Fig. 10). The results of PCA revealed a strong positive relationship between germination and CV-PBS/CV-MeOH, and strong negative relationships between germination and the ROS level as well as between germination (G) and the total antioxidants and non-protein antioxidants level in seeds (Fig. 10). The first two principal components accounted for 71.8% and 18.1% of the variance, respectively (89.9%). PCA loadings are provided in Supplementary Table 1.

## Discussion

Recalcitrant seeds are particularly susceptible to damage and loss of viability due to the overproduction and accumulation of ROS. This vulnerability arises from their less effective protective mechanisms compared to orthodox seeds [4, 6, 7, 10]. As a result, they serve as excellent models for studying the seed aging process along with its molecular and biochemical markers. Current research has focused on the recalcitrant nondormant seeds of *A. saccharinum*, which are characterized by active metabolism and rapid germination shortly after shedding [7]. To investigate the progress of viability decline, accelerated aging at 35 °C and ~ 45% of seed MC (Table 1) was applied to achieve a germination capacity curve in a timely manner. Under such storage stress conditions, which induced severe damage, seed germinability drastically declined from 96% in the control seeds (day 0) to only 1% after 14 days of aging. Furthermore, to determine the state of the redox milieu in seeds, the ROS levels were measured, since the free radical theory suggests that high levels of ROS play a key role in the mechanism of seed aging [4, 6, 12]. The ROS levels were tracked using the fluorescent probe H_2_DCFDA, which multiple oxidative agents oxidize, thus providing information on the oxidative stress level present in plant tissues [3, 53, 54]. As anticipated in previous reports [4, 12, 54], aging has manifested as the accumulation of ROS, with the greatest spikes occurring after 10 and 14 days, when the seeds presented 20% and 1% of germination capacity, respectively. ROS levels were negatively correlated with germination capacity (*R* = −0.86) and positively correlated with mean germination time (*R* = 0.88), demonstrating that an increase in ROS resulted in a decrease in total germination and prolonged germination time (Fig. 9).

The next steps involved performing two separate assessments of the seed antioxidant capacity to compare the techniques of the direct electrochemical technique (CV) with the CUPRAC-BCA assay. In general, the extraction yields, as well as the antioxidative and biological activities of extracts, are highly dependent on the chosen extraction technique and solvent type due to the diverse chemical properties, polarity, and solubility of bioactive compounds in plant material [55]. Because seeds from different species vary significantly in their storage material composition (oil-rich *versus* non-oil seeds), experimental testing appears to be necessary.

Therefore, to investigate the effectiveness of the CV technique, the seed extracts were prepared in two polar solvents: methanolic (80% MeOH) and 1x PBS (aqueous). It has already been demonstrated that extraction in highly polar solvents, such as water and methanol, are effective for plant material [55]. Additionally, the extraction procedure performed using 1x PBS was identical to that employed in CUPRAC-BCA, facilitating a direct comparison between the two analytical approaches. Moreover, 1x PBS extracts were previously used to obtain the electrochemical fingerprints of extracts derived from the leaves of various *Acer* species. The differences in the electrochemical fingerprint profiles were shown to distinguish different *Acer* species, perform a phylogenetic study, and identify the growth status in each season [35, 56, 57]. In the current study, the cyclic voltammograms revealed an anodic (positive) wave corresponding to the total oxidation potential of the analytes. As shown, the antioxidant capacity of the methanolic extract of the control seeds was greater than that of the aqueous extracts (Figs. 7 and 8), possibly due to the greater presence of phenolics, alkaloids, flavonoids, terpenoids and lipids with polar head groups reported to be more efficiently extracted in methanol than in aqueous solution or other organic solvents [55, 58, 59]. Indeed, methanol is typically more effective at extracting LMW polyphenols, which have strong antioxidant properties [48, 60]. However, in both cases, the results of the electrochemical measurements of the antioxidant capacity of the aqueous and methanolic extracts were highly correlated with total germination (Fig. 9, *R* = 0.86 and 0.92, respectively). To compare and validate the results obtained from CV experiments against those of other methods, measurements of both the reducing potential of antioxidants (Cu^2+^-AC) and non-protein antioxidants (Cu^2+^-NPAC) were conducted, along with measurements of glutathione disulfide (GSSG) and glutathione (GSH). Interestingly, the spectrophotometric analysis of extracts from seeds (CUPRAC-BCA) revealed results opposite to those recorded in the CV analyses, indicating an increase in the antioxidant status (Fig. 3). Previously, we successfully used this method to analyze the redox status in desiccated embryonic axes of sycamore (*Acer pseudoplatanus* L.*)* [43]. Here, we confirmed the accuracy of this method for embryonic axes isolated from *A. saccharinum* (Fig. 4). The differences in outcomes can be attributed to the fact that the applied spectrophotometric method does not directly measure antioxidative capacity, unlike the CV. Instead, it relies on transferring electrons from the reductants (antioxidants present in the sample) to a chemical oxidant (Cu^2+^) in the reaction, reducing Cu^2+^ to Cu^+^. However, it has been shown that Cu^+^ can decompose lipid peroxides by a Fenton reaction and initiates further radical chain reactions by donating the electron. Thus, Cu^2+^ is a catalyst in the presence of excessive antioxidants and returns to its oxidized form through a reaction with lipid peroxides [41]. Indeed, it has been shown that lipid peroxidation is one of the most widely reported metabolic changes related to seed deterioration and aging [61–63]. Moreover, the seeds (cotyledons) of *A. saccharinum* contain a significant amount of fatty acids, primarily comprising linoleic, oleic, palmitic, and cis-vaccenic acids [64, 65]. Consequently, the lipid-rich composition of *A. saccharinum* seeds makes the Cu^2+^-based method (CUPRAC-BCA) unsuitable for tracking changes in antioxidant capacity. This method is likely to be impractical for other lipid-rich plant matrices where spikes in ROS levels occur during progressive oxidative stress.

It has already been observed that the antioxidant capacity of biological samples cannot be accurately evaluated with a single assay, as many factors are often overlooked, and no universal assay exists that adequately measures antioxidant activity across all types of samples. For example, not all methods can be used to screen both lipophilic and hydrophilic samples [66]. As shown in the results, potential interferences within reaction mixtures can lead to misleading conclusions. It has also been previously reported that the correlations between CV measurements and other methods based on redox-active reagents are often inconsistent, particularly when comparing the CV and DPPH^•^ methods. CV has an advantage over DPPH^•^ radical methods since other less stable radicals of biological interest, such as ROO^•^ and OH^•^, exhibit higher potentials than the DPPH^•^ radical. Consequently, these differences may limit the effectiveness of the DPPH^•^ assay in accurately determining the true antioxidant capacity of certain biological samples [27]. Thus, the current research is not the first to report discrepancies between electrochemical and chemical methods. Importantly, due to the complexity of the plant material composition, separating each antioxidant compound and studying it individually is costly and inefficient, and overlooks potential synergistic interactions among these compounds. Additionally, a total antioxidant capacity assay using one individual chemical reaction seems unrealistic and reflects only the chemical reactivity under the specific conditions applied in that assay. Therefore, treating the data collected from chemical reactions with redox-active reagents for the general measurement of total antioxidant activity is misleading [24, 41, 66]. Therefore, CV seems to be a more reliable and versatile method for assessing total antioxidative capacity under various experimental conditions, regardless of the sample composition, the concentrations of the tested compounds, and crucially, the progress of oxidation damage in samples, such as in the case of aging seeds. Unlike techniques such as differential pulse voltammetry (DPV), the broad anodic wave observed in CV detects multiple antioxidants and a possible synergistic effect of the mixture, but reduces the ability to distinguish individual compounds. Consequently, it is best suited for assessing the overall redox environment in the tested samples. The limitation of the technique is the amount of biological sample needed for a single biological replicate. In the current study, approximately 1 mg of seed DW was used. This requirement could be challenging when working with small seeds or endangered species. Moreover, while CUPRAC assay can be used to measure thiol-type antioxidants such as glutathione and non-protein thiols [67], CV technique based on a glassy carbon electrode requires expensive electrode modification, complicated use of electrochemical sensors, or synthesis of a complex compound containing glutathione and Cu^2+^ [68]. Therefore, although reagent interference is prevented in CV, its inability to resolve thiols (such as GSH) shows a limitation of this technique, necessitating additional assays for comprehensive redox profiling. Additionally, more research is needed to optimize extraction methods, solvent compositions, and voltammetric parameters, as well as to confirm that CV functions as a universal assay for seeds from various species.

The final step of the investigation involved comparing the changes in glutathione (GSH) levels with the CV results. Since GSH and GSSG exhibited a low CV response and could not be examined using this methodology [29], the approach used for measuring GSH and GSSG offers an independent assessment of the seed redox milieu. GSH is a key water-soluble thiol in plants composed of γ-L-glutamyl-L-cysteinyl-glycine residues. The GSSG/2GSH couple is present in high concentrations in all organisms, being a major redox buffer. Previous studies have demonstrated that the GSSG/2GSH couple is crucial for several key seed quality characteristics, including viability and longevity. Research has shown that increasingly oxidizing intracellular environments, characterized by a transition of glutathione to its oxidized form (GSSG), are associated with seed mortality [14, 15, 69–71]. For instance, a loss of 50% seed viability coincided with a 50% loss of total glutathione (GSH + GSSG), and the half-cell reduction potential of glutathione (*E*_GSSG/2GSH_) shifted from − 180 to −160 mV. Notably, a decrease was observed in both dried orthodox seeds subjected to accelerated aging at a high temperature of 50 °C and desiccated recalcitrant seeds [14] and embryonic axes [71]. However, it is essential to remember that a specific thiol/disulfide oxidative shift in aged seeds depends on the state of the cytoplasm, meaning that in the glassy cytoplasm, the shift toward a more oxidative milieu is less pronounced than that in the fluid cytoplasm. This highlights that different aging mechanisms occur when seeds are aged under varying cytoplasmic conditions [72]. Moreover, for seeds of various species, the shift can be distinct. For instance, E_GSSG/2GSH_ values in non-stored seeds of *Phaseolus vulgaris* is − 155 mV, while in *Lactuca sativa* − 245 mV, and in seeds with no germinability the values are − 105 and − 208 mV, respectively [72]. *Eugenia stipitata* dead seeds had E_GSSG/2GSH_ values between − 195 and − 171 mV, and viable seeds had values between − 235 and − 215 mV [73].

Here, we have demonstrated that the GSH level (Fig. 5) increased after one day of accelerated aging, and this change is followed by a significant decrease until the final reduction after 10 and 14 days. A similar track of changes was observed for the GSSG levels. Generally, this sequence of changes applies to the aggregated pool of GSH and GSSG (G_total_). Importantly, we did not observe a proportional increase in GSSG. This concurs with previous reports showing that the aging of tomato seeds at 7.1% of MC at 60 °C resulted in a decrease in GSH. Nevertheless, no proportional increase in oxidized GSSG was detected [74]. The fact that the formation of GSSG could only partly account for the decrease in GSH, resulting in a loss of total glutathione, implies that GSH was lost by reactions other than the formation of GSSG [74]. Disulfides are indeed formed under mild oxidizing conditions, remain quite stable in a biological environment, and typically do not undergo further oxidation within cells [75]. However, under strong oxidation conditions, GSH and GSSG may produce compounds in which the oxidation state of the sulfur atom is higher than that of disulfides, e.g., sulfonic acids [74, 75], so the loss of total glutathione during seed aging may also be explained by the formation of oxidation products other than disulfides. Moreover, a transient increase in total glutathione (GSH + GSSG) after one day of accelerated aging could be interpreted as a response to a new external condition (high temperature), corresponding to the resistance phase of stress. In this phase, protection and/or repair mechanisms, including antioxidant defense, are activated to maintain seed viability and germinability [73, 76]. This finding is in agreement with the observed high total germination capacity of *A. saccharinum* seeds. However, the stress exhaustion phase eventually led to seed death, which coincided with the significantly decreased GSH content. Nevertheless, a significant decrease in the reducing force (GSH) and an increase in the glutathione half-cell reduction potential (E_GSSG/2GSH_) resulted from a shift from − 192.6 mV to −174.9 mV, which coincided with a reduction in the total antioxidant capacity measured by CV. Statistical analysis has revealed a positive correlation between the GSH level and CV antioxidant capacity (Fig. 9, *R* = 0.54 and 0.73 for CV-MeOH and CV-PBS, respectively).

The results of the multidimensional PCA indicated a strong positive relationship between CV-MeOH/CV-PBS and germination. In contrast, a strong negative relationship existed between germination and ROS levels (Fig. 10, Supplementary Table 1). Additionally, PCA revealed that measuring neither the glutathione half-cell reduction potential (EGSSG/2GSH) nor GSSG or GSH separately can explain the changes in germinability during the aging of *A. saccharinum* seeds (Fig. 10), probably due to a severe oxidative milieu that leads to higher oxidation states of sulfur. Additionally, the measurement of antioxidants based on the Cu^2+^ reduction method showed a similarly strong negative correlation with the germination of *A. saccharinum* seeds. This is attributed to the technical issues and misleading analysis results described above. These findings demonstrate that the CV technique is, in fact, more reliable than other tested techniques and has significant potential for measuring changes in antioxidant properties during seed aging-induced loss of viability.

## Conclusion

A loss of redox homeostasis caused by excessive ROS overproduction and a reduced capacity of the seed antioxidant system aligns with the decline in seed viability. For the first time, the current study evaluated the total antioxidant capacity of seeds using the CV technique and its relationship with seed viability. The CV has demonstrated itself as a rapid, cost-effective, and dependable technique that can be applied to measure antioxidant capacity in extracts obtained from entire seeds. CV can be integrated into high-throughput workflows with multiwell electrochemical plates for seed banks and stored seed viability monitoring, reducing assay time from hours to minutes. Additionally, it avoids the need to isolate embryonic axes, which can be tissue-damaging and time-consuming, even for experienced seed bank personnel. CV surpasses other conventional methods that rely on redox-active reagents, such as the Cu^2+^ reduction assay (CUPRAC-BCA), and is, therefore, advisable as an alternative to spectroscopic assays.

## Supplementary Information

Supplementary Material 1.

Supplementary Material 2.

Supplementary Material 3.

## Data Availability

All data generated or analyzed during this study are included in this published article or its supplementary information files. The raw data are available from the corresponding author upon reasonable request.
