# Supplementary figures and images for: Cyclic voltammetry as a method for determining the viability of seeds: a case study on silver maple (Acer saccharinum L.)
Source: BMC Plant Biol. 2025 Aug 14;25:1074. doi: 10.1186/s12870-025-07137-x (PMC12355840; doi:10.1186/s12870-025-07137-x)

Supplementary Data 1. Seed of *Acer saccharinum* L. (silver maple)

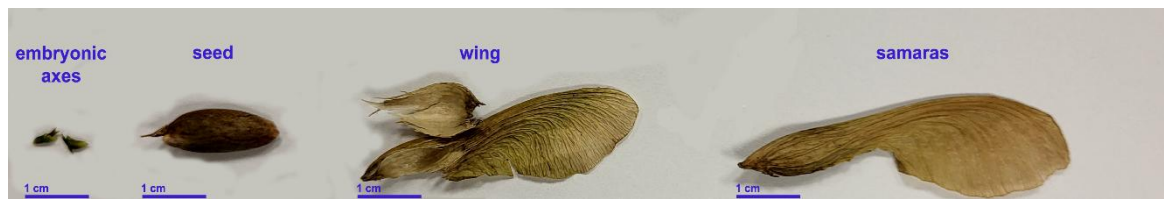

Supplement: Supplementary file 1 — Supplementary Material 1. [file 12870_2025_7137_MOESM1_ESM.pdf]
