# Supplementary material for: Cyclic voltammetry as a method for determining the viability of seeds: a case study on silver maple (Acer saccharinum L.)
Source: BMC Plant Biol. 2025 Aug 14;25:1074. doi: 10.1186/s12870-025-07137-x (PMC12355840; doi:10.1186/s12870-025-07137-x)
Supplement: Supplementary file 2 — Supplementary Material 2. [file 12870_2025_7137_MOESM2_ESM.pdf]

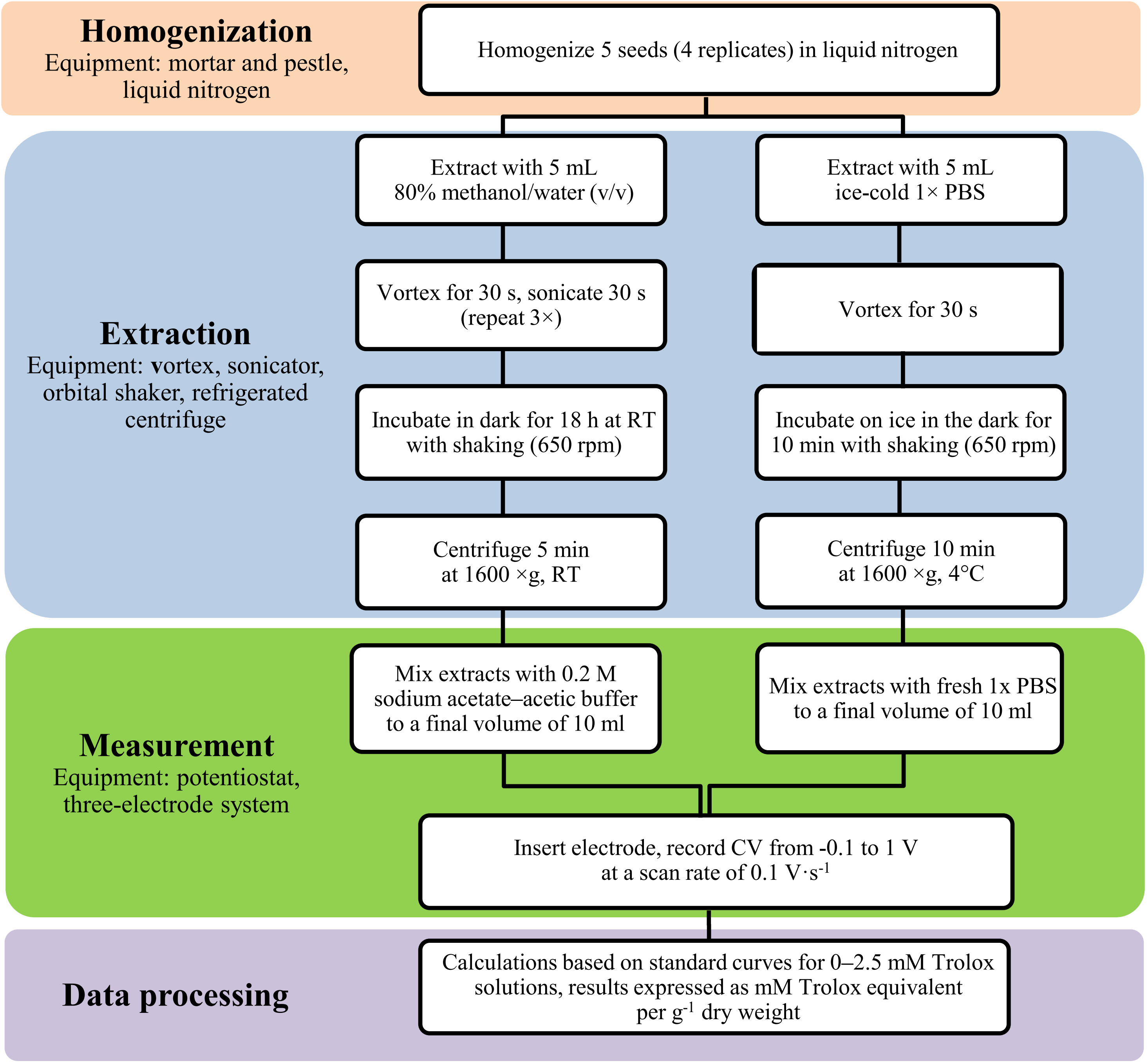

Homogenization
Equipment: mortar and pestle, liquid nitrogen
Homogenize 5 seeds (4 replicates) in liquid nitrogen
Extraction
Equipment: vortex, sonicator, orbital shaker, refrigerated centrifuge
Extract with 5 mL 80% methanol/water (v/v)
Extract with 5 mL ice-cold 1× PBS
Vortex for 30 s, sonicate 30 s (repeat 3×)
Vortex for 30 s
Incubate in dark for 18 h at RT with shaking (650 rpm)
Incubate on ice in the dark for 10 min with shaking (650 rpm)
Centrifuge 5 min at 1600 ×g, RT
Centrifuge 10 min at 1600 ×g, 4°C
Measurement
Equipment: potentiostat, three-electrode system
Mix extracts with 0.2 M sodium acetate–acetic buffer to a final volume of 10 ml
Mix extracts with fresh 1x PBS to a final volume of 10 ml
Insert electrode, record CV from -0.1 to 1 V at a scan rate of 0.1 V·s-1
Data processing
Calculations based on standard curves for 0–2.5 mM Trolox solutions, results expressed as mM Trolox equivalent per g-1 dry weight
