# Supplementary material for: Cyclic voltammetry as a method for determining the viability of seeds: a case study on silver maple (Acer saccharinum L.)
Source: BMC Plant Biol. 2025 Aug 14;25:1074. doi: 10.1186/s12870-025-07137-x (PMC12355840; doi:10.1186/s12870-025-07137-x)
Supplement: Supplementary file 3 — Supplementary Material 3. [file 12870_2025_7137_MOESM3_ESM.pdf]

Supplementary Table 1.

Supplementary Table 1a. Coordinates of principal component analysis

	Dim.1	Dim.2	Dim.3	Dim.4	Dim.5	Dim.6	Dim.7	Dim.8
G	-0.93773	0.302512	-0.05885	-0.02084	-0.04152	0.048329	-0.0923	0.112553
CV-PBS	-0.92	0.246367	0.02971	0.131404	0.244881	0.091823	0.079204	0.008611
CV-MeOH	-0.6597	0.589785	-0.42603	-0.14595	0.065134	-0.07543	-0.03931	-0.05168
GSSG	-0.8016	-0.5827	-0.08109	-0.03686	0.022099	-0.08749	0.032436	0.027304
totalG	-0.78289	-0.53188	-0.19644	-0.1689	-0.08881	0.16599	0.004707	-0.04018
GSH	-0.79876	-0.58561	-0.07719	-0.02646	0.030081	-0.0961	0.037242	0.028853
ROS	0.769299	-0.32013	-0.39782	0.381658	0.013072	0.022869	-0.03152	0.009081
AC	0.933906	-0.26382	-0.03372	-0.18804	0.139463	0.017371	-0.04025	0.018742
NPAC	0.933906	-0.26382	-0.03372	-0.18804	0.139463	0.017371	-0.04025	0.018742
E	0.887524	0.323601	-0.24826	-0.13547	-0.07775	0.014719	0.134358	0.057303

Supplementary Table 1b. Contribution of principal component analysis

	Dim.1	Dim.2	Dim.3	Dim.4	Dim.5	Dim.6	Dim.7	Dim.8
G	12.24997	5.050898	0.754198	0.142698	1.432476	3.752739	20.73646	55.86986
CV-PBS	11.79118	3.350018	0.19225	5.673886	49.83531	13.54649	15.26874	0.327024
CV-Me	6.062709	19.19865	39.53091	6.999513	3.525703	9.140542	3.761271	11.7802
GSSG	8.951432	18.73989	1.432248	0.446459	0.405871	12.29687	2.560704	3.287916
totalG	8.538356	15.61379	8.404732	9.374239	6.555103	44.26797	0.053936	7.119772
GSH	8.8881	18.92778	1.297828	0.230108	0.751967	14.8374	3.375817	3.671506
ROS	8.244554	5.656339	34.46923	47.86473	0.141997	0.840272	2.41789	0.363678
AC	12.1502	3.841476	0.247585	11.61892	16.16382	0.484828	3.94378	1.549083
NPAC	12.1502	3.841476	0.247585	11.61892	16.16382	0.484828	3.94378	1.549083
E	10.97331	5.779689	13.42344	6.030529	5.02394	0.34807	43.93762	14.48187

Supplementary Table 1c. Loadings of principal component analysis

	Comp.1	Comp.2	Comp.3	Comp.4	Comp.5	Comp.6	Comp.7	Comp.8
G	0.34999956	0.22474203	0.086845	0.03777539	0.119686	0.19372	0.455373	0.747461
CV-PBS	0.34338282	0.18303053	-0.04385	-0.23819921	-0.70594	0.368056	-0.39075	0.057186
CV-MeOH	0.2462257	0.4381626	0.628736	0.26456592	-0.18777	-0.30233	0.19394	-0.34322
totalG	0.29918943	-0.4328959	0.119677	0.06681757	-0.06371	-0.35067	-0.16002	0.181326
GSSG	0.29220465	-0.3951429	0.289909	0.30617379	0.256029	0.665342	-0.02322	-0.26683
GSH	0.29812917	-0.4350607	0.113922	0.04796958	-0.08672	-0.38519	-0.18373	0.191612
ROS	-0.28713332	-0.2378306	0.587105	-0.6918434	-0.03768	0.091666	0.155496	0.060306
AC	-0.34857134	-0.1959968	0.049758	0.34086537	-0.40204	0.06963	0.19859	0.124462
NPAC	-0.34857134	-0.1959968	0.049758	0.34086537	-0.40204	0.06963	0.19859	0.124462
E	-0.33125983	0.24040985	0.36638	0.24557136	0.224141	0.058997	-0.66285	0.380551
